# Preparation of a MoS_2_/carbon nanotube composite as an electrode material for high-performance supercapacitors†

**DOI:** 10.1039/c8ra05158e

**Published:** 2018-08-20

**Authors:** Xiaobo Chen, Jingguo Ding, Jing Jiang, Guoce Zhuang, Zhihai Zhang, Peizhi Yang

**Affiliations:** School of New Energy and Electronic Engineering, Yancheng Teachers University Yancheng 224051 P. R. China chenxbok@126.com; Key Laboratory of Education Ministry for Advance Technique and Preparation of Renewable Energy Materials, Institute of Solar Energy, Yunnan Normal University Kunming 650500 P. R. China pzhyang@hotmail.com

## Abstract

MoS_2_ and MoS_2_/carbon allotrope (MoS_2_/C) composites for use as anodes in supercapacitors were prepared *via* a facile hydrothermal method. In this study, we report the effects of various carbon-based materials (2D graphene nanosheet (GNS), 1D carbon nanotube (CNT), and 0D nano carbon (NC)) on the electrochemical performances. Among all nanocomposites studied, MoS_2_/CNT exhibited the best electrochemical performance. Specifically, the MoS_2_/CNT composite exhibits remarkable performances with a high specific capacitance of 402 F g^−1^ at a current density of 1 A g^−1^ and an outstanding cycling stability with 81.9% capacitance retention after 10 000 continuous charge–discharge cycles at a high current density of 1 A g^−1^, making it adaptive for high-performance supercapacitors. The superiority of MoS_2_/CNT was investigated by field emission scanning electron microscopy and transmission electron microscopy, which showed that MoS_2_ nanosheets were uniformly loaded into the three-dimensional interconnected network of nanotubes, providing an excellent three dimensional charge transfer network and electrolyte diffusion channels while effectively buffering the collapse and aggregation of active materials during charge–discharge processes. Overall, the MoS_2_/CNT nanocomposite synthesized by a simple hydrothermal process presents a new and promising candidate for high-performance anodes for supercapacitors.

## Abstract 2

The effect of carbon supports on the electrochemical performance of MoS_2_ nanosheets for supercapacitor applications was investigated.

## Graphical abstract

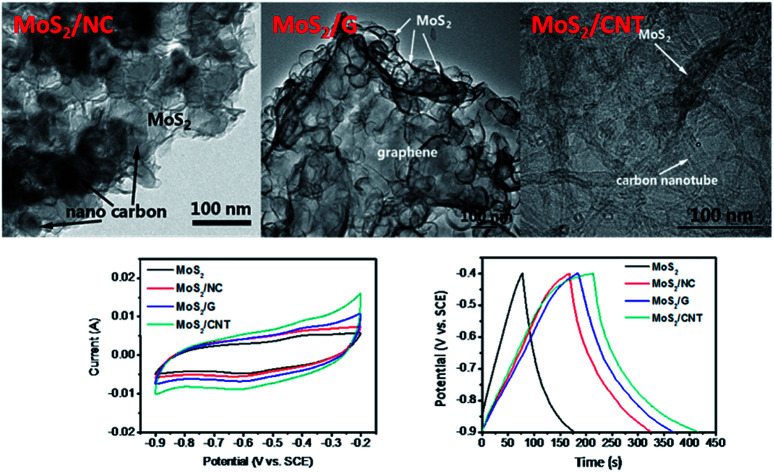

## 1. Introduction

There has been an ever-increasing and urgent demand for developing renewable energy resources and electrical energy storage technologies in response to the increasing issues of environmental pollution and energy consumption. Among various energy storage devices, supercapacitors (SCs) are considered as one of the most promising devices for energy storage due to high power density, high energy density, long lifespan, quick charging/discharging.^1^ There are three factors that are most commonly considered in performance assessment of SCs: electrode material, electrolyte and SCs device architecture.^2^ Among which, the electrode material plays an important role in determining the performance of SCs, so it is still very attractive to develop and design a novel electrode material with prominent electrochemical properties.

Two-dimensional (2D) layered MoS_2_ nanosheets may perform best for applications in electrochemical energy storage because of their large surface area and exposed active sites.^3,4^ Several recent reports have been focused on the supercapacitor performance of pure MoS_2_ with diverse morphologies. For examples, Huang *et al.* reported that MoS_2_ nanosheets as electrode materials exhibited a specific capacitance up to 129.2 F g^−1^ at 1 A g^−1^ with capacitance retention of 85.1% after 500 cycles;^5^ Wang *et al.* prepared flower-like MoS_2_ which showed a specific capacitance of 168 F g^−1^ at a current density of 1 A g^−1^ when applied as electrode materials for electrochemical capacitors.^6^ These results indicate that MoS_2_ is a promising electrode material for supercapacitors owing to its two-dimensional sheets-like morphology, which can provide large surface area for double-layer charge storage. In addition, the easy diffusion of electrolyte into the inner region (interlayers) of the MoS_2_ electrode at lower scan rates can provide faradaic capacitance, which acts a good part in improved charge storage capability.^7^ These studies on the supercapacitive properties of pure MoS_2_ showed only moderate performance as an electrode material due to the relatively poor electronic conductivity and stability. Therefore, the electrochemical properties of MoS_2_ can be improved *via* making composites with other electroactive materials, such as carbon allotrope materials and conductive polymer to overcome the above problem.^4,7–10^ Various kinds of carbon materials have been suggested as conductivity supporters (or additives) for MoS_2_, such as nano carbon black,^10–12^ graphene,^3,4,13^ and carbon nanotube.^8,14,15^ However, up to present, the comparative investigation about the effect of carbon supporters on the electrochemical performance for supercapacitor application is still rarely reported.

Herein, to combine all these merits and enhance the electrochemical performance of MoS_2_, a straightforward hydrothermal method has been developed in this work to synthesize MoS_2_/NC, MoS_2_/G and MoS_2_/CNT composites through hydrothermal method. The morphology, surface areas, porous structures and electrochemical properties of as-prepared MoS_2_/NC, MoS_2_/G and MoS_2_/CNT composites had been studied comparatively.

## 2. Experimental

### 2.1. Material synthesis

In this work, graphene nanosheets (GNSs), carbon nanotubes (CNT) and nano carbon black (NCB) are purchased from Tanfeng Tech. Inc (Suzhou, China). Other reagents are analytical grade without further purification and purchased from Zhanyun Chemical Co., Ltd., Shanghai, China.

MoS_2_/C composites (MoS_2_/NC, MoS_2_/G and MoS_2_/CNT) were prepared through hydrothermal method. Firstly, 30 mg carbon based nanomaterials (NC, G, or CNT) were firstly blended with 20 mL ethanol and ultrasonicated for 1 h to obtain a solution A (carbon based nanomaterials dispersion). Secondly, 1 mmol Na_2_MoO_4_·2H_2_O and 5 mmol thiourea were dissolved in 60 mL of DI water and added to the above dispersion and stirred for 1 h. The reaction mixture was then transferred into a Teflon-lined autoclave, heated up to 210 °C, and maintained at this temperature for 24 h. The resultant black precipitate was centrifuged, washed three times with DI water, followed by ethanol washing, and dried under vacuum. For comparison, the pure MoS_2_ was also prepared *via* the same method without carbon.

### 2.2. Materials characterizations

The crystallographic structure was characterized by X-ray diffraction (XRD) on an X-ray powder diffractometer (Smartlab-9 X-ray diffractometer, Rigaku, Japan) using Cu Kα radiation (*λ* = 1.5418 Å). The specific surface areas (BET method) and porous structures measurements were performed in a model ASAP 2020 Micromeritics apparatus at 77 K. Field emission scanning electron microscopic (FESEM) images were carried out by Zeiss Supra 35VP, transmission electron microscopic (TEM) and high-resolution transmission electron microscopic (HRTEM) images were observed by FEI Tecnai F20.

The electrodes were obtained by mixing active material (85 wt%), acetylene black (10 wt%) and polytetrafluoroethylene (PTFE, 60 wt% dispersion in water) binder (5 wt%). Then, it was dried in hot air oven at 80 °C for 0.5 h, and pressed under the pressure of 10 MPa and finally dried at 100 °C for 12 h. Each electrode contained about 5 mg of active material.

### 2.3. Electrochemical measurements

The electrochemical properties were tested using three-electrode system with the working electrode, Pt counter electrode and Hg/HgO electrode as the reference electrode in the 1 M Na_2_SO_4_ aqueous solution at room temperature. Cyclic voltammetry (CV) measurements were performed on a CHI660E electrochemical workstation (CHI, Shanghai China) and galvanostatic charge–discharge (GCD) curves were measurements by a Neware battery testing workstation (Neware, Shengzhen China). Electrochemical impedance spectroscopy (EIS) was performed on the electrochemical work-station (CHI660D) by applying an ac amplitude of 0.5 mV in the frequency from 10^6^ to 10^−2^ Hz.

## 3. Result and discussion

### 3.1. Structure characterizations

**Fig. 1:**
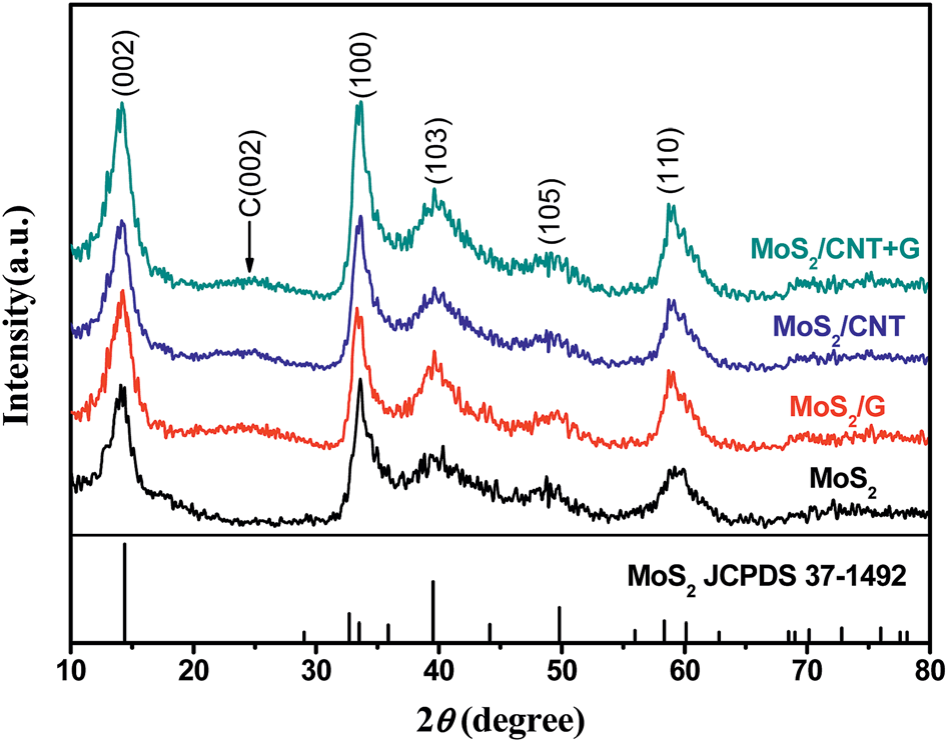
XRD patterns of MoS_2_, MoS_2_/NC, MoS_2_/G and MoS_2_/CNT.

Fig. 1 shows XRD patterns of pure MoS_2_ and MoS_2_/NC, MoS_2_/G and MoS_2_/CNT composites. From the XRD pattern of as-prepared MoS_2_ nanosheets, the peaks of 14.4°, 32.7°, 39.5° and 58.3° are in good accordance with the standard values of MoS_2_ (JCPDS card no. 37-1492). In terms of MoS_2_/NC, MoS_2_/G and MoS_2_/CNT composites, a broad diffraction hump between 21° and 27° could be attributed to the (002) reflection of a graphitic structure, suggesting the existence of carbon-based materials. In addition, no characteristic peaks of other impurities have been detected, implying that the MoS_2_ and MoS_2_/NC, MoS_2_/G and MoS_2_/CNT composites are of high purity.

### 3.2. Morphology characterizations

**Fig. 2:**
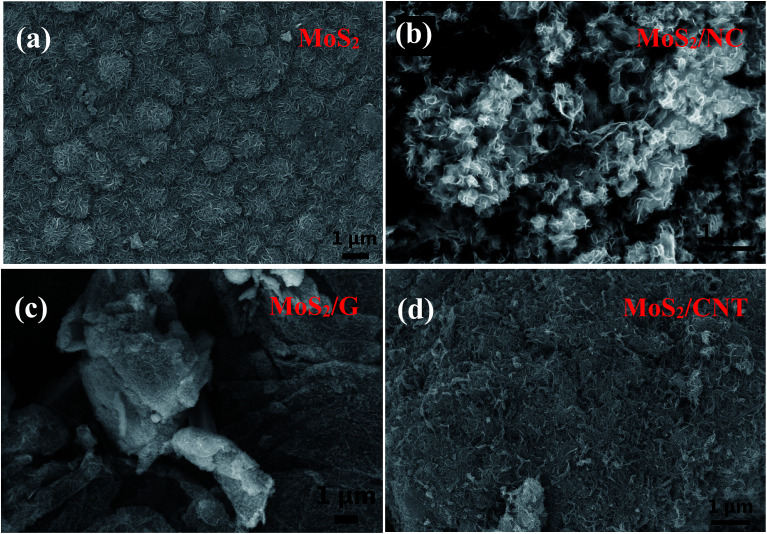
FESEM images of (a) MoS_2_, (b) MoS_2_/NC, (c) MoS_2_/G and (d) MoS_2_/CNT composites.

The shape and morphology of the hydrothermally synthesized samples were investigated by SEM and TEM images. The distinctive FE-SEM morphologies of MoS_2_, MoS_2_/NC, MoS_2_/G and MoS_2_/CNT are displayed in Fig. 2 and S2.† The FE-SEM micrograph of pure MoS_2_ (Fig. 2(a)) suggested that few layered 2D MoS_2_ hierarchical nanosheets are aggregated to form microspheres with the size of 1 μm. The MoS_2_/NCs show a nanoflower structure (Fig. 2(b)). There is no agglomeration of free nano carbon particles in the composite, implying nano carbon particles are intercalated in the interlayers of the MoS_2_ nanosheets. Fig. 2(c) shows the SEM image of the MoS_2_/G. It can be observed that the graphene nanosheets are decorated randomly with MoS_2_ nanosheets. Incorporation of CNTs can effectively reduce the stacking and yield highly separated ultrathin MoS_2_ nanosheets (Fig. 2(d)), which are later determined by TEM. More importantly, the relatively long CNTs form interconnected network to facilitate the fast electron transport. It is clearly observed that CNTs interweaved with MoS_2_ nanosheets. This unique architecture is anticipated to dramatically increase the electronic conductivity and maintain structural integrity thus to boost the rate performance and cycling stability in supercapacitor applications.

**Fig. 3:**
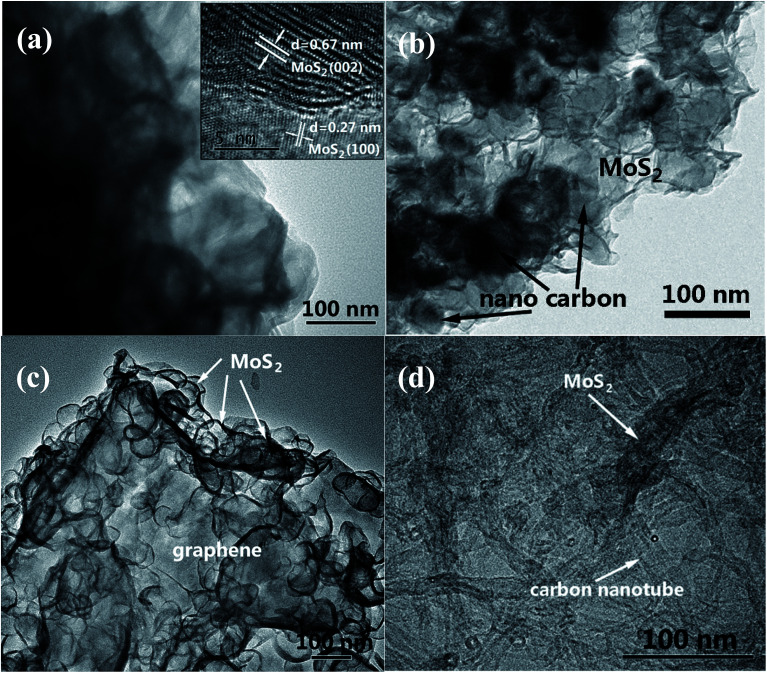
TEM images of (a) MoS_2_, (b) MoS_2_/NC, (c) MoS_2_/G and (d) MoS_2_/CNT composites.

**Fig. 4:**
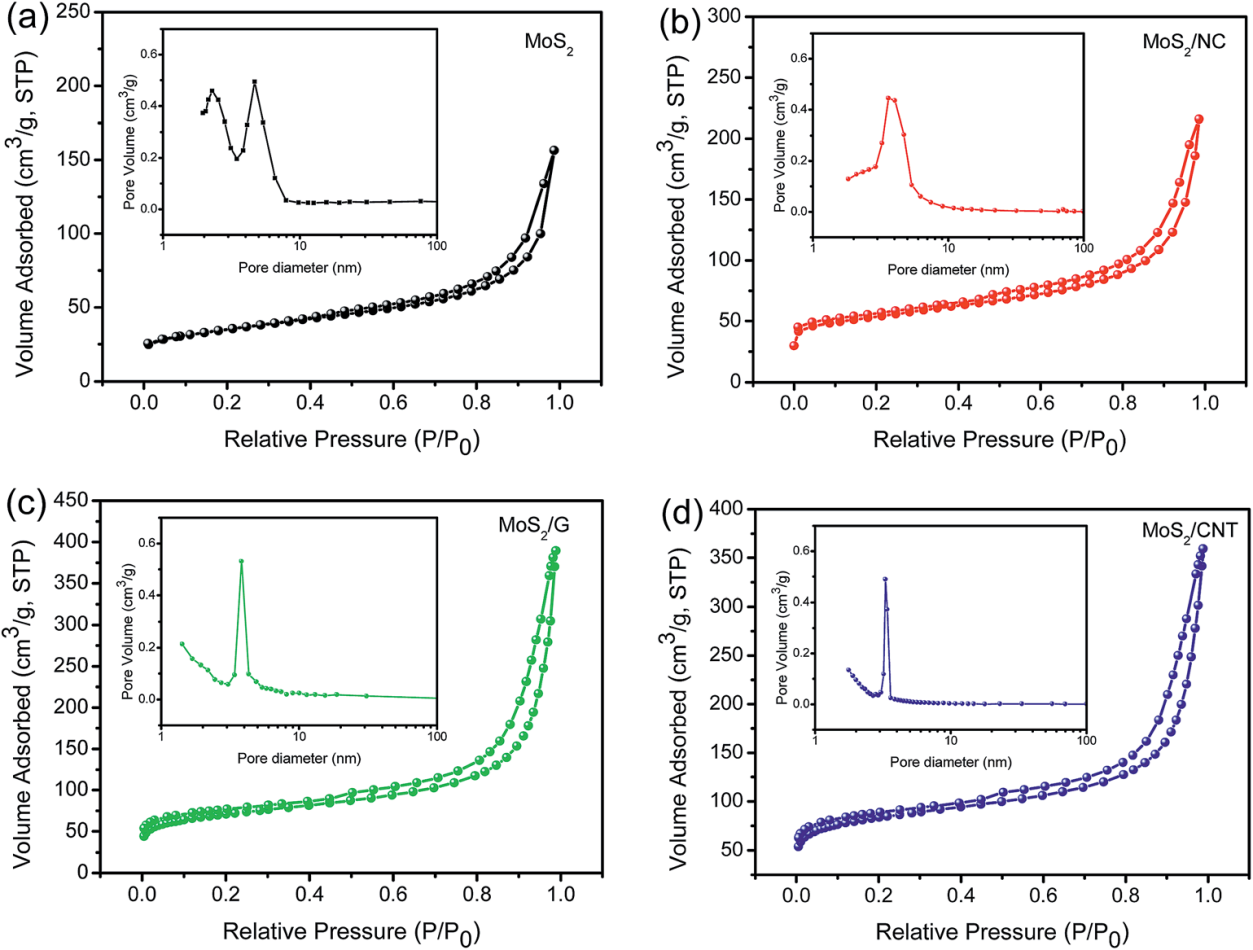
The N_2_ adsorption–desorption isotherms of MoS_2_ (a), MoS_2_/NC (b), MoS_2_/G (c) and MoS_2_/CNT composites (d). The insets show corresponding pore size distributions.

As shown in Fig. 3(a), the TEM image of MoS_2_ forms two-dimensional nanosheets. The high-resolution TEM (HRTEM) image in the inset of Fig. 3(a) indicates the lattice figure with interlayer spacing of 0.67 nm which corresponds to the (002) plane of MoS_2_.^16^ Also, the lattice spacing of 0.27 nm which belongs to the (100) plane of MoS_2_ can be observed in the same image.^17^ SEM image of MoS_2_/NC (Fig. 3(b)) displays that the MoS_2_ shell coats nano carbon tightly. It can be also seen that GS are decorated randomly by MoS_2_ nanosheets to generate MoS_2_/G composite in Fig. 3(c). Fig. 4(d) shows more evidence of the extreme porous nature of the nanohybrid composed of ultrathin MoS_2_ nanosheets supported/separated by CNTs.

Furthermore, the synthesized MoS_2_/CNT was also characterized by EDS. From Fig. S1(a),† the signals of C, O, S and Mo elements can be seen. From the observation of the four element mappings of Mo, S, C and O (Fig. S1(b)†), the elements are uniformly distributed throughout the MoS_2_/CNT composite. Therefore, the HRTEM and EDS results indicated that the MoS_2_/carbon hybrid has been successfully synthesized.

Fig. 4 shows the N_2_ adsorption–desorption isotherms and corresponding pore size distribution curves of samples. The isotherms of as-prepared materials all exhibit the type IV hysteresis loop as defined by IUPAC. The BET surface areas of the MoS_2_, MoS_2_/NC, MoS_2_/G and MoS_2_/CNT are 116, 219, 236 and 275 m^2^ g^−1^, respectively. Obviously, the surface area of MoS_2_/CNT is 2.4 times as much as pure MoS_2_, attributing to the compact interconnected network structure, which not only provides large surface area for charge storage but also facilitates electrolyte penetration through the mesopores. The pore size distributions of these samples are shown in the insets, confirming that the samples have mesoporous characteristics. The well-developed 3D interconnected porous network with multilevel pores can provide not only favorable transport channels for electrolyte but also strong mechanical strength. Based on the above mentioned merits, the MoS_2_/CNT composite can be employed as an excellent electrode material for SCs and the detailed electrochemical measurements are performed as follows.

### 3.3. Electrochemical performances

**Fig. 5:**
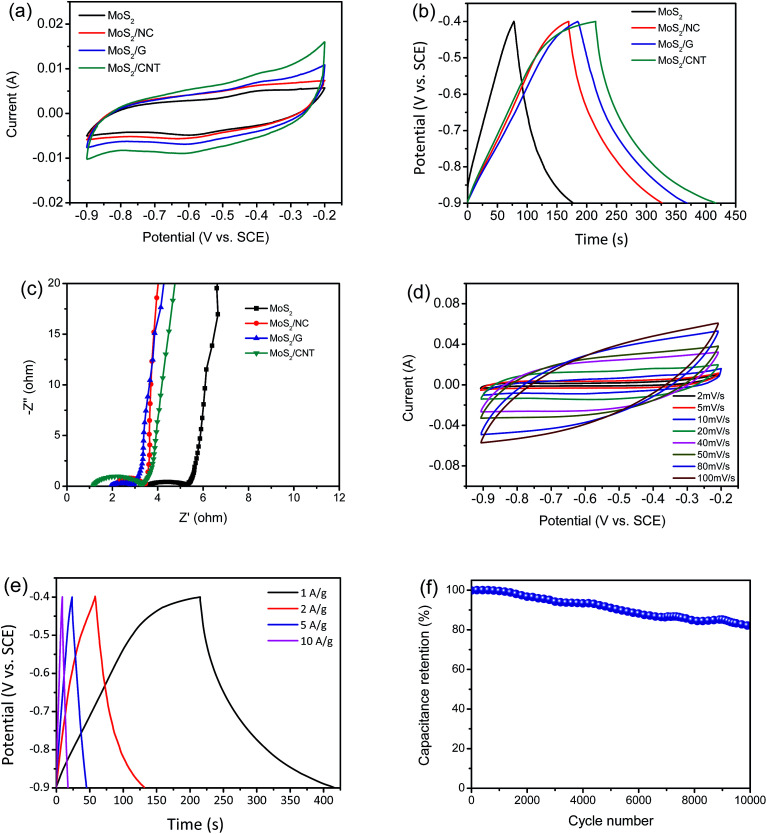
Electrochemical characterizations of MoS_2_, MoS_2_/NC, MoS_2_/G and MoS_2_/CNT composites. (a) CV curves of samples at a scan rate of 10 mV s^−1^; (b) GCD curves of samples at a current density of 1 A g^−1^; (c) Nyquist plots; (d) CV and (e) GCD curves of MoS_2_/CNT in 1 M Na_2_SO_4_ electrolyte at various current densities and voltage scan rates, respectively. (f) Specific capacitance of MoS_2_/CNT composite *versus* cycle number at the current density of 1 A g^−1^.

Both CV and GCD measurements are carried out to survey the electrochemical properties. As shown in Fig. 5(a), the CV curves of MoS_2_ and MoS_2_/NC, MoS_2_/G and MoS_2_/CNT composites are performed within a potential window from 0.9 to 0.2 V at a scanning rate of 10 mV s^−1^ in 1 M Na_2_SO_4_. The measured voltammograms of MoS_2_ and MoS_2_/NC, MoS_2_/G and MoS_2_/CNT show quasi-rectangular shapes with a pair of small humps at *ca.* −0.6 V and *ca.* −0.4 V, which represent intercalation and expulsion of Na^+^ ions between the MoS_2_ layers and can be represented as follows:^3^

1 MoS_2_ + Na^+^ + e^−^ ⇌ MoS − SNa

The output current of MoS_2_/CNT is larger than those of MoS_2_ and MoS_2_/NC and MoS_2_/G. One major weakness of pure MoS_2_ based SCs is its inherent resistivity.^3,4^ The results indicate that the conductivity of MoS_2_ can be improved by combining MoS_2_ and CNT together to form composites, meanwhile, the specific capacitance also increases. Fig. 5(b) shows the GCD curves of MoS_2_ and MoS_2_/NC, MoS_2_/G and MoS_2_/CNT at a current density of 1 A g^−1^ with voltage between −0.9 and −0.4 V. It is clear that the discharge time of MoS_2_/CNT is much longer compared to MoS_2_, MoS_2_/NC and MoS_2_/G. The specific capacitances can be calculated using the eqn (2):

2 *C*_s_ = *I* × Δ*t*/(*m* × Δ*V*)

where *I* is the constant discharge current (A), *m* is the mass of active materials in electrode (g) and Δ*t* is the discharge time (s) in the potential window Δ*V* (V).^18^ The specific capacitances of MoS_2_, MoS_2_/NC, MoS_2_/G and MoS_2_/CNT are 194, 316, 360 and 402 F g^−1^ at 1 A g^−1^, respectively, which is in agreement with the result of the CV curves. This exhibits the excellently electrochemical activity of MoS_2_/CNT composite. To further understand the excellent electrochemical performances of MoS_2_/CNT, the EIS measurements for samples of MoS_2_, MoS_2_/NC, MoS_2_/G and MoS_2_/CNT are carried out (Fig. 5(c)). Obviously, the equivalent series resistance (*R*_ESR_, the total resistance of ionic resistance of electrolyte, intrinsic resistance of substrate, and contact resistance at the interface of the active material/current collector) of the MoS_2_/CNT (1.19 Ω) is smaller than those of the pure MoS_2_ (3.28 Ω), MoS_2_/NC (1.99 Ω), and MoS_2_/G (1.37 Ω). The lowest *R*_ESR_ value for MoS_2_/CNT indicates the improved electrical conductivity of the MoS_2_/CNT composite material, attributing to the short transport path of ions and charges of porous structure as well as the effective contact between MoS_2_ and CNT.

CV measurement is carried out to further investigate the charge storage mechanism of MoS_2_/CNT in the three electrode system. Fig. 5(d) shows CV curve area of MoS_2_/CNT increases with increasing scan rates (from 2 to 100 mV s^−1^). The CV loop of MoS_2_/CNT electrode at 100 mV s^−1^ is still close to rectangular, suggesting that the MoS_2_/CNT composite can be employed as an excellent electrode material. It should be emphasized that the curves for the initial cycles at lower scan rates exhibit faradaic capacitance in addition to electric double layer capacitance, due to diffusion of the ions into the MoS_2_ interlayers at low scan rates,^19^ improving charge storage capabilities. The specific capacitances of the MoS_2_/CNT calculated from the GCD curves (Fig. 5(e)) are 402, 292, 215 and 172 F g^−1^ at current densities of 1, 2, 5 and 10 A g^−1^, respectively. Since such a decrease of specific capacitances is common with increasing scan rates, and caused by the difficulty of ion diffusion and the insufficient faradaic redox reaction at high scan rates.^4^

The cycle stability of MoS_2_/CNT composite is important for its practical applications. The cycling performance of MoS_2_/CNT at a current density of 1 A g^−1^ was shown in Fig. 5(f). Importantly, the specific capacitance still maintains 81.9% level after 10 000 consecutive cycles. Particularly, there is no clear capacitance drop occurred over 1000 cycles (99.6% retention) due to the support between CNT and MoS_2_ nanosheets, presenting excellent cycling stability of MoS_2_/CNT. More interestingly, compared with other MoS_2_/carbon composites with electrolyte of 1 M Na_2_SO_4_, MoS_2_/CNT showed higher specific capacitance and more excellent electrochemical stability than those of most reported MoS_2_/carbon composite electrode (Table 1). The electrochemical reversibility can be further explained by EIS measurements (Fig. S3†). Noticeably, the EIS spectra are similar in terms of the curve shape except that a moderate change of equivalent series resistance. Before and after 10 000 cycles, the *R*_ESR_ values of MoS_2_/CNT electrode are 1.19 Ω and 1.62 Ω, respectively, indicating that the MoS_2_/CNT electrode has a relatively good stability. We further capture the SEM image of the hierarchical MoS_2_/CNT composite after 10 000 cycles in Fig. S4.† The possible reason can be found in Fig. S4,† the composite after 10 000 cycles with somewhat aggregation (highlighted by red circles), thus leading to decrease of the capacity.

**Table 1:** Comparison on electrochemical properties of MoS_2_/carbon composites with electrolyte of 1 M Na_2_SO_4_^a^

Electrode materials	Preparation method	Maximum specific capacitance	Electrolyte	Cyclic ability	Ref.
MoS_2_/graphene	Solution phase exfoliation	11 mF cm^−2^ at 5 mV s^−1^	1 M Na_2_SO_4_	250% at 1 mA cm^−2^ after 10 000 cycles	3
MoS_2_/graphene aerogel	Lithium intercalation and hydrothermal assembly	268 F g^−1^ at 0.5 A g^−1^	1 M Na_2_SO_4_	93% at 1 A g^−1^ after 1000 cycles	20
MoS_2_/graphene hybrid films	Layer-by-layer assembly	282 F g^−1^ at 20 mV s^−1^	1 M Na_2_SO_4_	93% at 2 A g^−1^ after 1000 cycles	21
MoS_2_/MWCNT	Hydrothermal method	452.7 F g^−1^ at 1 A g^−1^	1 M Na_2_SO_4_	95.8% at 1 A g^−1^ after 1000 cycles	8
MoS_2_/C	Hydrothermal method	201.4 F g^−1^ at 0.2 A g^−1^	1 M Na_2_SO_4_	94.1% at 1.6 A g^−1^ after 1000 cycles	10
MoS_2_/carbon aerogel	Hydrothermal method	260 F g^−1^ at 1 A g^−1^	1 M Na_2_SO_4_	92.4% at 1 A g^−1^ after 1500 cycles	22
MoS_2_ nanoflowers/3DG	Hydrothermal method	410 F g^−1^ at 1 A g^−1^	1 M Na_2_SO_4_	113.6% at 2 A g^−1^ after 1000 cycles	4
MoS_2_/CNT	Hydrothermal method	402 F g^−1^ at 1 A g^−1^	1 M Na_2_SO_4_	99.6% at 1 A g^−1^ after 1000 cycles, 81.9% at 1 A g^−1^ after 10 000 cycles	This work

a Abbreviations: MWCNT – multi-walled carbon nanotube; C – carbon; 3DG – three-dimensional grapheme; GS – graphene sheets; CNT – carbon nanotube.

## 4. Conclusions

In summary, the MoS_2_/C composites were successfully synthesized *via* a facile hydro-thermal method with carbonaceous materials (GNSs, CNTs, and CB). The as-prepared MoS_2_/C composites showed superior electrochemical performances than that of bare MoS_2_ counterpart when used as an anode in supercapacitors. Especially, the MoS_2_/CNT exhibited more excellent than those of most reported MoS_2_/carbon composite electrode. The MoS_2_/CNT composite exhibited remarkable performances with a high specific capacitance of 402 F g^−1^ at a current density of 1 A g^−1^ and an outstanding cycling stability with 81.9% capacitance retention after 10 000 continuous charge–discharge cycles at a high current density of 1 A g^−1^, making it desirable for high-performance supercapacitors. FESEM and TEM analysis confirmed that MoS_2_ nanosheets have been loaded into the 3-D network of CNT. The 3-D network structure could buffer the volume changes of MoS_2_ nanosheets at high current densities and maintain high electrical conductivity. Hence, the MoS_2_/CNT composite material is a promising candidate for electrode of SCs.

## Conflicts of interest

The authors declare no conflict of interest.

## Supplementary Material

RA-008-C8RA05158E-s001

## References

[cit1] Burke A. (2000). J. Power Sources.

[cit2] Yan J., Wang Q., Wei T., Fan Z. (2014). Adv. Energy Mater..

[cit3] Bissett M. A., Kinloch I. A., Dryfe R. A. W. (2015). ACS Appl. Mater. Interfaces.

[cit4] Sun T., Li Z., Liu X., Ma L., Wang J., Yang S. (2016). J. Power Sources.

[cit5] Huang K. J., Zhang J. Z., Shi G. W., Liu Y. M. (2014). Electrochim. Acta.

[cit6] Wang X., Ding J., Yao S., Wu X., Feng Q., Wang Z., Geng B. (2014). J. Mater. Chem. A.

[cit7] Ma G., Peng H., Mu J., Huang H., Zhou X., Lei Z. (2013). J. Power Sources.

[cit8] Huang K. J., Wang L., Zhang J. Z., Wang L. L., Mo Y. P. (2014). Energy.

[cit9] Tang H., Wang J., Yin H., Zhao H., Wang D., Tang Z. (2015). Adv. Mater..

[cit10] Fan L. Q., Liu G. J., Zhang C. Y., Wu J. H., Wei Y. L. (2015). Int. J. Hydrogen Energy.

[cit11] Weng Q., Wang X., Wang X., Zhang C., Jiang X., Bando Y., Golberg D. (2015). J. Mater. Chem. A.

[cit12] Zhang S., Hu R., Dai P., Yu X., Ding Z., Wu M., Li G., Ma Y., Tu C. (2016). Appl. Surf. Sci..

[cit13] Firmiano E. G. D. S., Rabelo A. C., Dalmaschio C. J., Pinheiro A. N., Pereira E. C., Schreiner W. H., Leite E. R. (2014). Adv. Energy Mater..

[cit14] Chen M., Dai Y., Wang J., Wang Q., Wang Y., Cheng X., Yan X. (2017). J. Alloys Compd..

[cit15] Wang S., Zhu J., Shao Y., Li W., Wu Y., Zhang L., Hao X. (2017). Chem.–Eur. J..

[cit16] Huang Z., Zhang L., Li M., Ran W., Lu Y., Yang B., Long Z. (2017). Nanosci. Nanotechnol. Lett..

[cit17] Liu X., Xing Z., Zhang H., Wang W., Zhang Y., Li Z., Wu X., Yu X., Zhou W. (2016). ChemSusChem.

[cit18] Qian W., Chen Z., Cottingham S., Merrill W. A., Swartz N. A., Goforth A. M., Clare T. L., Jiao J. (2012). Green Chem..

[cit19] Khawula T. (2016). J. Mater. Chem. A.

[cit20] Yang M., Jeong J. M., Huh Y. S., Choi B. G. (2015). Compos. Sci. Technol..

[cit21] Patil S., Harle A., Sathaye S., Patil K. (2014). CrystEngComm.

[cit22] Huang K. J., Wang L., Zhang J. Z., Xing K. (2015). J. Electroanal. Chem..

