# Supplementary material for: Preparation of a MoS2/carbon nanotube composite as an electrode material for high-performance supercapacitors
Source: RSC Adv. 2018 Aug 20;8(52):29488–94. doi: 10.1039/c8ra05158e (PMC9085153; doi:10.1039/c8ra05158e)
Supplement: RA-008-C8RA05158E-s001 [file RA-008-C8RA05158E-s001.pdf]

Electronic Supplementary Material (ESI) for RSC Advances.
This journal is © The Royal Society of Chemistry 2018

# Supporting Information

## Preparation of $MoS_2$/carbon allotrope composite as electrode material for high-performance supercapacitors

Xiaobo Chen [1,*] Jingguo Ding [1], Jing Jiang [1], Guoce Zhuang [1], Zhihai Zhang [1], Peizhi Yang [2,*]

1 School of New Energy and Electronic Engineering, Yancheng Teachers University, Yancheng, 224051, PR China

2 Key Laboratory of Education Ministry for Advance Technique and Preparation of Renewable Energy Materials, Institute of Solar Energy, Yunnan Normal University, Kunming, 650500, PR China

E-mail addresses: chenxbok@126.com (X. Chen); pzhyang@hotmail.com (P. Yang)

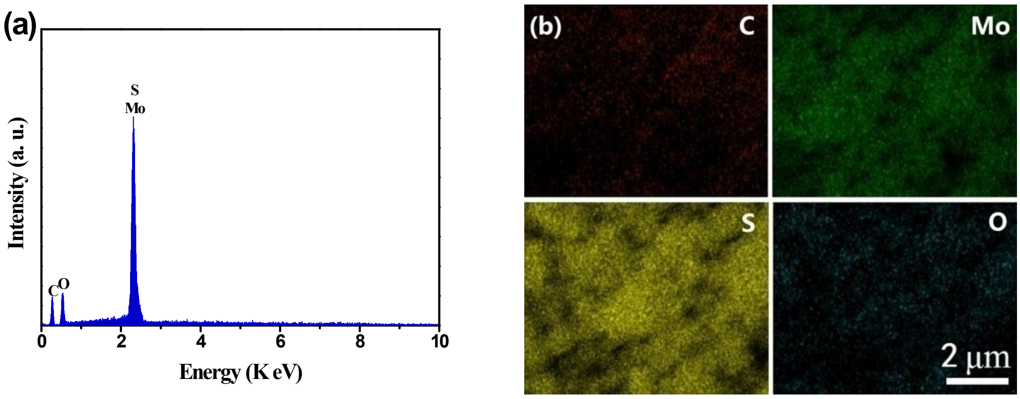


**Fig. S1** EDS pattern (a) and elemental mapping (b) of $MoS_2$-CNT.

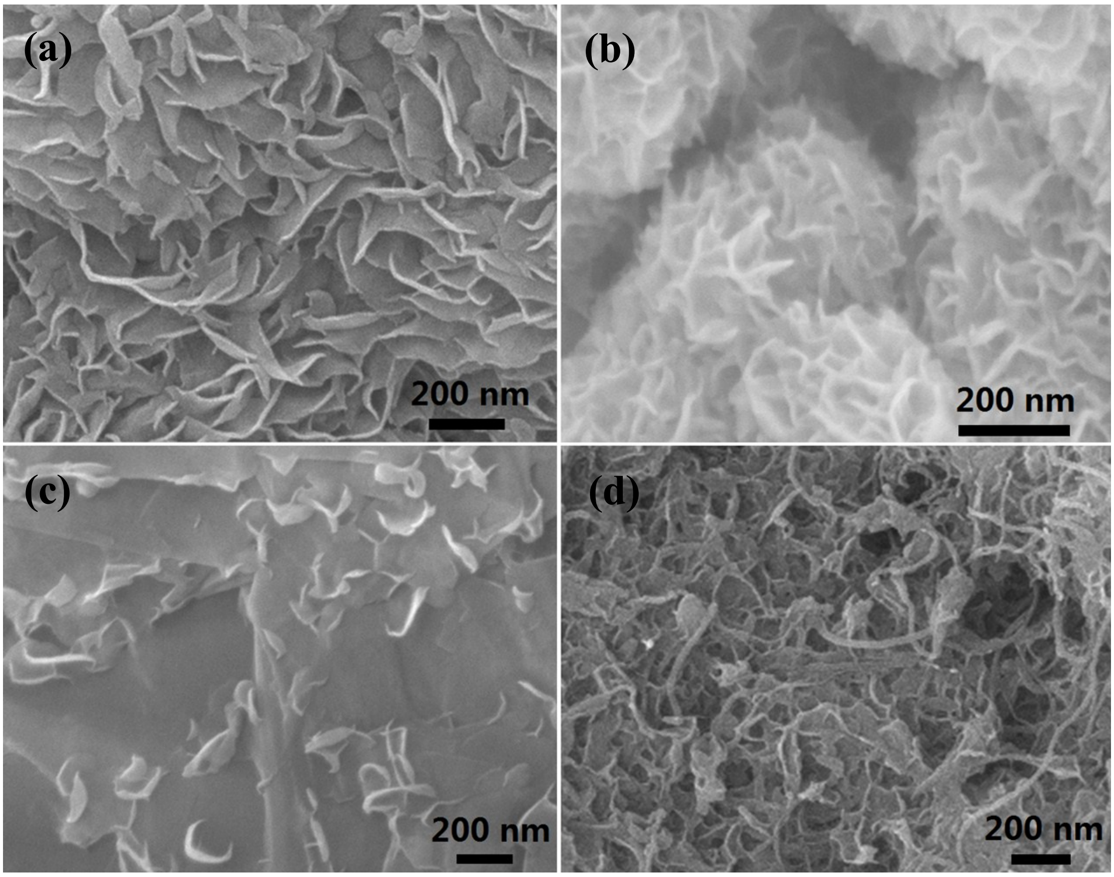


**Fig. S2** FESEM images of (a) $MoS_2$, (b) $MoS_2$/NC, (c) $MoS_2$/G and (d) $MoS_2$/CNT composites.

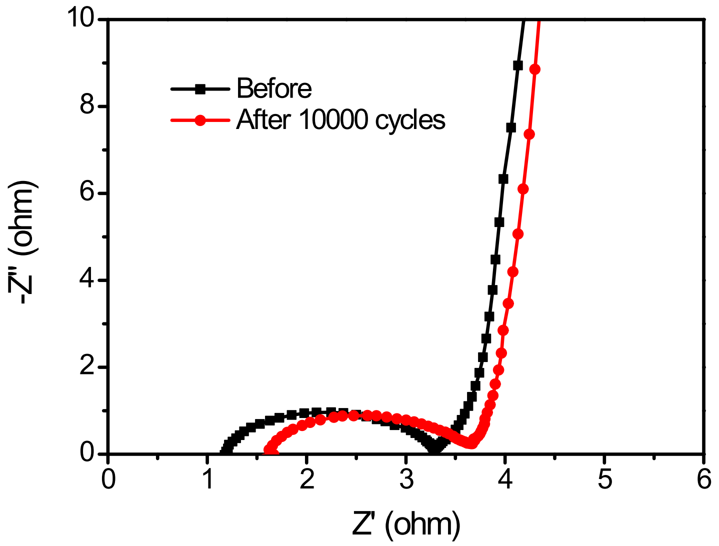


**Fig. S3** Nyquist plot of 3D $MoS_2$/CMG before and after the 10000 cycles cycling.

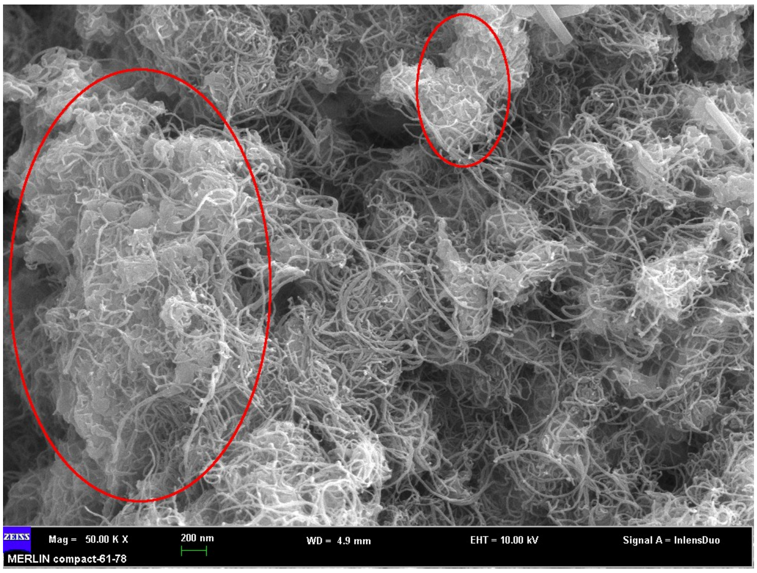


**Fig. S4** SEM image of 3D $MoS_2$/CMG after the 10000 cycles cycling.
